# Prevalence and clinical, social, and health care predictors of miscarriage

**DOI:** 10.1186/s12884-021-03682-z

**Published:** 2021-03-05

**Authors:** Erin Strumpf, Ariella Lang, Nichole Austin, Shelley A. Derksen, James M. Bolton, Marni D. Brownell, Dan Chateau, Patricia Gregory, Maureen I. Heaman

**Affiliations:** 1 Department of Epidemiology, Biostatistics, and Occupational Health, McGill University Leacock Building, Room 418, 855 Sherbrooke Street West, Montreal, QC H3A 2T7 Canada; 2 Department of Economics, McGill University, Montreal, Canada; 3 School of Nursing, McGill University, Montreal, Canada; 4 Manitoba Centre for Health Policy, University of Manitoba, Winnipeg, Canada; 5 Department of Psychiatry, University of Manitoba, Winnipeg, Canada; 6 Department of Community Health Sciences, University of Manitoba, Winnipeg, Canada; 7 Department of Nursing, Red River College, Winnipeg, Canada; 8 College of Nursing, University of Manitoba, Winnipeg, Canada; 9 Department of Obstetrics, Gynecology and Reproductive Sciences, University of Manitoba, Winnipeg, Canada

**Keywords:** Miscarriage, Health services, Women’s health, Manitoba

## Abstract

**Background:**

Pregnancy loss is common and several factors (e.g. chromosomal anomalies, parental age) are known to increase the risk of occurrence. However, much existing research focuses on recurrent loss; comparatively little is known about the predictors of a first miscarriage. Our objective was to estimate the population-level prevalence of miscarriages and to assess the contributions of clinical, social, and health care use factors as predictors of the first detected occurrence of these losses.

**Methods:**

In this population-based cohort study, we used linked administrative health data to estimate annual rates of miscarriage in the Manitoba population from 2003 to 2014, as a share of identified pregnancies. We compared the unadjusted associations between clinical, social, and health care use factors and first detected miscarriage compared with a live birth. We estimated multivariable generalized linear models to assess whether risk factors were associated with first detected miscarriage controlling for other predictors.

**Results:**

We estimated an average annual miscarriage rate of 11.3%. In our final sample (*n* = 79,978 women), the fully-adjusted model indicated that use of infertility drugs was associated with a 4 percentage point higher risk of miscarriage (95% CI 0.02, 0.06) and a past suicide attempt with a 3 percentage point higher risk (95% CI -0.002, 0.07). Women with high morbidity were twice as likely to experience a miscarriage compared to women with low morbidity (RD = 0.12, 95% CI 0.09, 0.15). Women on income assistance had a 3 percentage point lower risk (95% CI -0.04, -0.02).

**Conclusions:**

We estimate that 1 in 9 pregnant women in Manitoba experience and seek care for a miscarriage. After adjusting for clinical factors, past health care use and morbidity contribute important additional information about the risk of first detected miscarriage. Social factors may also be informative.

**Supplementary Information:**

The online version contains supplementary material available at 10.1186/s12884-021-03682-z.

## Background

Spontaneous abortion, also known as early pregnancy loss and miscarriage, typically occurs in the first trimester of gestation [1]. Depending on the data source, these losses are estimated to occur in 10–25% of clinically-recognized pregnancies in developed countries [2, 3]. Approximately 50% of early-term miscarriages are attributed to chromosomal abnormalities – “random numeric chromosome errors” [2] – which influences the conventional wisdom that early-term pregnancy losses occur at random, and that all women are at risk [4]. However, other maternal (and sometimes paternal) characteristics have been identified as risk factors, particularly for recurrent miscarriages, including clinical [5–9], social [10, 11], behavioral [6, 12, 13], and health factors [9, 14–16].

Reliable statistics on both the population prevalence and distribution of these losses early in pregnancy are challenging to estimate. The loss may occur before the woman knows she was pregnant and whether a woman seeks medical attention will depend on the accessibility of services, severity of symptoms, and the woman’s circumstances. Published estimates range from 5% [11] to 52% [17] of pregnancies, with the bulk of the estimates between 13 and 30% [7, 10, 16, 18, 19]. This variation is due in part to small samples [8], as well as different populations and time periods under study (e.g. upward trends over time are partly due to to increased awareness of pregnancy [18]). In prospective studies, estimates are affected by the specificity and sensitivity of different methods and assays used to detect pregnancy [19] and when in their menstrual cycle or gestation women are enrolled in the study [20]. Retrospective studies use diagnostic codes to identify miscarriages among hospitalized women and interview data for non-hospitalized women [6, 21], where recall bias can be a particular challenge [20]. Using existing, population-level data that includes both inpatient and outpatient health care utilization may be a cost-effective way to estimate the prevalence of these events.

Two important gaps remain in the literature regarding the frequency and distribution of these pregnancy losses. First, relatively little is known about the prevalence of losses early in pregnancy at the population level. From a health care system planning perspective it would be particularly useful to know the prevalence of miscarriages for which the woman seeks medical care in either an inpatient or outpatient setting. Second, although studies have explored the predictors of miscarriage, much of this research has addressed risk factors for recurrent miscarriages (which occur in only 1–3% of couples [20]). The predictors of first losses, or those affecting approximately 1 in 5 pregnancies, are not as well understood. Furthermore, a population-wide assessment of predictors of losses early in pregnancy, including clinical, social, and health care use risk factors, has not yet been conducted to our knowledge.

To address these gaps, we use administrative health data to identify women who experience and seek care for a miscarriage. We estimate annual rates of these events in the Manitoba population from 2003 to 2014, and then describe the clinical, social, and health care use profiles of women who experience their first^1^ miscarriage early in pregnancy compared with women who have a live birth. Our results help us assess what characteristics are associated with first miscarriage at a population level and will help inform and better prepare clinicians and staff.

## Methods

### Data

The Population Research Data Repository at the Manitoba Centre for Health Policy, University of Manitoba, contains linkable, de-identified administrative data on all residents of Manitoba registered under the universal, public Manitoba Health Services Insurance Plan [22]. The data are based on information contained in the Manitoba Health Services Insurance Plan Registry and from health insurance claims routinely filed by physicians and health care facilities with Manitoba Health [23]. Several studies have found these administrative health data to have a high degree of reliability and validity [24, 25]. The Repository data include the provincial health insurance registry, fee-for-service physician billings, hospital discharge abstracts, emergency department visits, pharmaceutical prescriptions, individual sociodemographics, and vital statistics, all linked using an encrypted Personal Health Identification Number. Area-level sociodemographics come from the Canadian census.

About 13,500–16,000 births occur in the province each year [26]. Most pregnant women receive their prenatal care from a FP/family physician (~ 40%) or an OBGYN (~ 40%) [26]. About 70% deliver with an OBGYN, 25% deliver with a FP/family physician, and home births are uncommon (< 1%) [26], which suggests that the relevant population is captured in the Repository databases. In this population-based cohort study, we extracted data from 1984 to 2014 and estimated rates of miscarriage from 2003 to 2014.

### Exposure definition and analysis

Women were considered exposed if they experienced (and sought treatment for, given our reliance on administrative data) a miscarriage during the study period. We identified miscarriages based on ICD diagnostic codes in the hospital and physician billing data, and chief complaint codes in the ED data. Women were coded as having a miscarriage if they had at least one hospitalization or physician visit with a diagnosis of non-induced abortion or at least one ED visit with a chief complaint of “pregnancy issues” (either < or > 20 weeks) and no delivery (live or stillborn) in the following 40 weeks (Additional file 1). The administrative data do not contain information on gestational age for pregnancies that do not result in a live birth, but given how clinicians use these diagnostic codes, we are reasonably confident we are capturing losses earlier than 20 weeks’ gestation.

To estimate population rates of pregnancy loss due to miscarriage, we calculated the number of these events as a share of women identified as pregnant in each calendar year. We included first and subsequent losses in the numerator. The denominator of all pregnancies includes all deliveries (live and stillborn, singleton and multiple births), ectopic pregnancies, miscarriages, and therapeutic abortions (Additional file 2). Because the same health event for a woman may appear in multiple data sources on different dates, we considered the same diagnosis in different data sources within +/− 90 days to be the same event.

To compare the characteristics of women who experienced a miscarriage with those who did not, we narrowed our sample to compare women who experienced their *first* miscarriage (observable in our data) with women who had a singleton live birth (and no previous losses, including but not limited to losses due to prior miscarriage or ectopic pregnancy) between 2003 and 2012 (Additional file 3). Again, our exposure definition relies upon interaction with the healthcare system and includes women who experienced and sought care for a miscarriage for the first time (we revisit this in the Discussion section). Women in both groups may have had previous live births.

We compared these two groups across a number of clinical, social, and health care use factors measured before the loss/birth. Some are established risk factors for loss early in pregnancy, while others are potential risk factors that we are uniquely able to investigate given our linked population databases (see Additional file 4 for a full list of variable definitions). We compared means and distributions of each of these variables separately to understand their association with experiencing a loss. Given our large sample size, we also calculated standardized differences to assess meaningful differences in characteristics between groups. Standardized differences compare the difference in means in units of the pooled standard deviation and are independent of sample size (a value > 10% indicates a meaningful difference [27]).

We estimated generalized linear multivariable regression models (binomial, identity link) to assess whether known or potential risk factors are independently associated with experiencing a loss after controlling for other predictors. In Model 1, we controlled for calendar year and include the group of clinical factors listed in Additional file 4. Model 2 added social factors to Model 1, and Model 3 contained health care use, social, and clinical factors. Due to the high correlation between use and cost variables, we included only costs related to hospitalization, ambulatory care, and psychotropic prescriptions.

The study was approved by the Human Health Research Ethics Board at the University of Manitoba, the Manitoba Health Information Privacy Committee, and the McGill University Faculty of Medicine Research Ethics Board. All analyses were conducted using SAS. Resource Utilization Bands (RUB), which are a measure morbidity based on health care utilization patterns, were created using The Johns Hopkins Adjusted Clinical Group® (ACG®) Case-Mix System version 11.

## Results

**Fig. 1:**
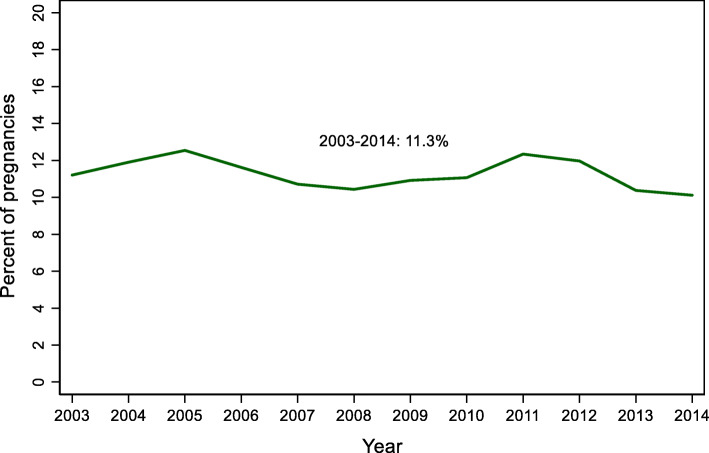
Prevalence of miscarriage, Manitoba (2003–2014)

Figure 1 shows the annual rate of miscarriages in Manitoba from 2003 to 2014. Using our exposure definition we identify 2217 to 2787 miscarriages per year, or 10.1 to 12.5% of pregnancies. These rates are quite consistent over the 10-year period but increase slightly in the last few years. The addition of losses identified using emergency department data from 2009 to 12 increases the number of miscarriages by 1916 (or 6.6% of all miscarriages) in those years.

**Fig. 2:**
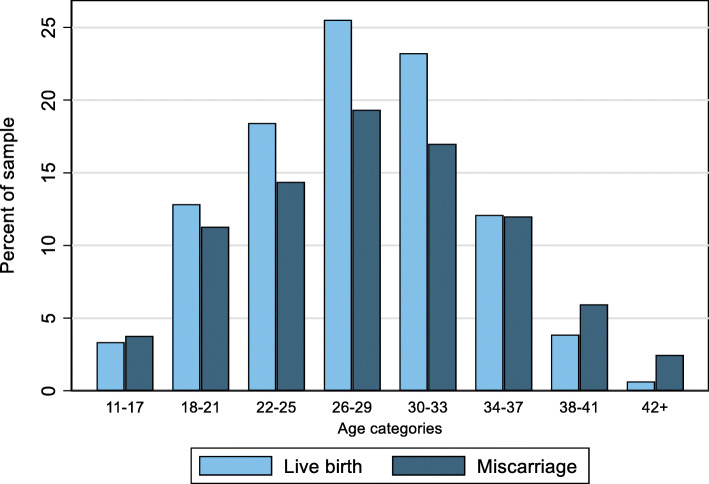
Maternal age at miscarriage or live birth

Table 1 presents the bivariate comparisons of clinical, social, and health care use factors between women who experience and seek care for their first miscarriage (*n* = 11,231) and women who have a live birth and no previous recorded losses (*n* = 68,747). Women who experienced their first miscarriage were more likely to be nulliparous (48% vs. 42%, Std. Diff = 11.54) than women with a live birth. Women who experienced their first miscarriage were more likely to have had mood or anxiety disorders in the two years before the loss, relative to women with a live birth (16% vs. 12%, Std. Diff. = 11.18). Women with a loss were slightly older on average than women with a live birth (28.5 vs. 27.8 years, Std. Diff. = 10.58). Figure 2 illustrates that first losses are more prevalent at younger and older ages. Rates of diagnosed chronic diseases, use of infertility drugs, and substance abuse were similar between the two groups.

**Table 1:** Bivariate comparison of women who experience their first miscarriage versus live birth^a^

Characteristic	Miscarriage (***n*** = 11,231)	Live birth (***n*** = 68,747)	Std. diff.
	Mean/proportion ± SD		
**Clinical**			
Previous c-section^b^	0.12 ± 0.32	0.12 ± 0.32	−0.45
Diabetes^c^	0.04 ± 0.20	0.03 ± 0.18	4.44
Endometriosis^c^	0.01 ± 0.09	0.01 ± 0.08	3.46
Hypertension^d^	0.03 ± 0.18	0.03 ± 0.17	1.74
Infertility drug use^e^	0.04 ± 0.19	0.02 ± 0.15	7.45
Mood or anxiety disorders^e^	0.16 ± 0.37	0.12 ± 0.33	11.18
Substance abuse^e^	0.03 ± 0.17	0.03 ± 0.16	1.53
Suicide attempt^f^	0.01 ± 0.10	0.01 ± 0.08	3.98
Mother’s age at event	28.47 ± 6.82	27.80 ± 5.79	10.58
Parity^b,g^			
Nulliparous	0.48 ± 0.50	0.42 ± 0.49	11.54
Primiparous	0.29 ± 0.45	0.33 ± 0.47	−8.72
Multiparous	0.23 ± 0.42	0.25 ± 0.43	−3.99
**Social**			
Mother’s SEFI^h^	0.23 ± 1.13	0.18 ± 1.11	3.96
Income assistance^i^	0.09 ± 0.28	0.10 ± 0.30	4.58
**Health care use**			
Morbidity (RUB)^d^	2.30 ± 0.94	2.11 ± 1.00	19.73
Hospitalization costs (adjusted)^d,j^	177 ± 1614	84 ± 1070	6.77
Hospitalization LOS (in days)^d^	0.17 ± 2.33	0.08 ± 1.31	5.1
Amb. phys. (FP) costs (adjusted)^d,j^	140 ± 136	94 ± 103	38.64
Amb. phys. (FP) visits^d^	4.21 ± 4.16	2.88 ± 3.10	36.15
Any incident psychotropic Rx^k^	0.06 ± 0.23	0.05 ± 0.22	2.75
Number of psychotropic Rx^k^	0.85 ± 4.94	0.66 ± 5.48	3.57
Psychotropic Rx costs (adjusted)^j,k^	33 ± 184	26 ± 159	4.27

*Std. diff* Standardized difference, *RUB* Resource Utilization Band, *SEFI* Socioeconomic Factor Index, *Amb. phys* Ambulatory physician, *Rx* prescription, *LOS* Length of stay

Data are presented as means (for continuous variables) or proportions (for binary variables) ± SD

^a^Sample excludes women not covered for at least 2 years prior to event; ^b^Since 1984; ^c^In the 3 years before event; ^d^In the year before event; ^e^In the 2 years before event; ^f^In the 5 years before event; ^g^0 = nulliparous, 1 = primiparous, 2 = multiparous; ^h^At time of event; ^i^For at least one month in the year before event; ^j^In 2010 dollars; ^k^Over a 1-year period starting 2 years before the event date

**Fig. 3:**
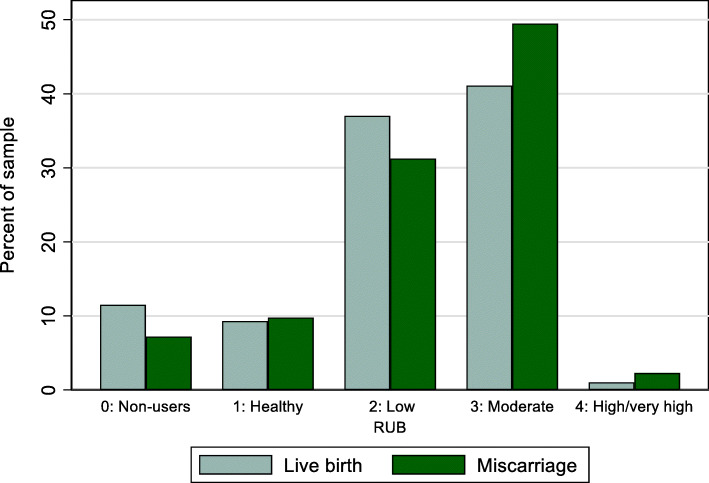
Maternal morbidity (RUB) in the year before the miscarriage or live birth

Women who experienced their first loss had higher predicted morbidity in the year before the event compared to women with a live birth (average RUB 2.30 vs. 2.11, Std. Diff. = 19.73). Figure 3 shows that women who experience their first miscarriage are more likely to be in the moderate (49.5% vs. 41.1%) or high/very high (2.3% vs. 1.0%) predicted morbidity categories in the year before the event, and are less likely to be non-users of health care services or to have low predicted morbidity than women who have a live birth. They also had more family physician visits in an outpatient setting (4.21 vs. 2.88, Std. Diff. = 36.15) and higher costs associated with outpatient family physician care ($140 vs. $942,010 Canadian dollars, Std. Diff. = 38.64) in the year before the loss.

The first multivariable model estimates conditional associations between first recorded miscarriage and clinical risk factors (Table 2). All models control for maternal age; models 2 and 3 additionally control for maternal region of residence (Additional files 5 and 6 present the full set of coefficients and global tests of statistical significance, respectively). Conditional on maternal age, primiparous and multiparous women are 3 percentage points less likely (95% CIs − 0.04, − 0.02; − 0.03, − 0.02) than nulliparous women to experience a miscarriage relative to a live birth. Relative to the average miscarriage rate during the study period of 11.3%, this translates to a 26.5% lower rate. Women who used prescription infertility treatment are 4 percentage points more likely (95% CI 0.02, 0.06) to experience a miscarriage. This elevated risk is of a similar magnitude as women in their late 30s compared to a 29-year old woman (e.g., age 37 RD = 0.05, 95% CI 0.03, 0.07). Women who experienced mood or anxiety disorders are 4 percentage points (95% CI 0.03, 0.05), or 33%, more likely to experience a miscarriage. Women who attempted suicide are 5 percentage points more likely (95% CI 0.02, 0.08) to experience a miscarriage.

**Table 2:** Multivariable analysis of women who experience their first miscarriage versus live birth^*^

Variable	Model 1: RD (95%CI) ***n*** = 79,978	Model 2: RD (95%CI) ***n*** = 79,846	Model 3: RD (95%CI) ***n*** = 79,846
Year of event	−0.0002 (− 0.001, 0.001)	−0.0001 (0.001, 0.001)	− 0.000 (− 0.001, 0.001)
**Clinical**			
Parity^a^			
Primiparous vs. nulliparous	−0.03 (− 0.04, − 0.02)	−0.03 (− 0.03, − 0.02)	−0.02 (− 0.03, − 0.02)
Multiparous vs. nulliparous	−0.03 (− 0.03, − 0.02)	−0.03 (− 0.03, − 0.02)	−0.02 (− 0.03, − 0.02)
Previous c-section^a^	0.01 (0.001, 0.016)	0.01 (0.001, 0.02)	0.01 (− 0.002, 0.01)
Diabetes^b^	0.02 (0.01, 0.03)	0.02 (0.002, 0.03)	0.004 (−0.01, 0.02)
Endometriosis^b^	0.03 (−0.002, 0.07)	0.03 (0.0002, 0.07)	0.02 (−0.01 0.06)
Hypertension^c^	−0.01 (− 0.02, 0.01)	−0.01 (− 0.02, 0.008)	−0.02 (− 0.03, − 0.004)
Infertility drug use^d^	0.04 (0.02, 0.06)	0.04 (0.03, 0.06)	0.04 (0.02, 0.06)
Mood or anxiety disorders^d^	0.04 (0.03, 0.05)	0.04 (0.03, 0.05)	0.02 (0.02, 0.03)
Substance abuse^d^	0.004 (−0.01, 0.02)	0.002 (−0.01, 0.02)	− 0.01 (− 0.03, 0.01)
Suicide attempt^e^	0.05 (0.02, 0.08)	0.04 (0.01, 0.07)	0.03 (−0.002, 0.07)
**Social** ^**f, g**^			
Mother’s SEFI^h^		0.01 (0.004, 0.01)	0.01 (0.005, 0.01)
Income assistance^i^		−0.02 (−0.03, − 0.02)	−0.03 (− 0.04, − 0.02)
**Health care use**			
Morbidity (RUB)^c^			
0 vs. 2			−0.03 (− 0.04, − 0.03)
1 vs. 2			0.03 (0.02, 0.03)
3 vs. 2			0.04 (0.03, 0.04)
4+ vs. 2			0.12 (0.09, 0.15)
Hospitalization costs (2010$)^c, j^			0.004 (0.003, 0.006)
Amb. phys. Costs (2010$)^c, j^			0.01 (−0.02, 0.03)
Psychotropic Rx costs (2010$)^k, l^			−0.002 (− 0.004, 0.0002)

*RD* risk difference, *CI* confidence interval, *RUB* Resource Utilization Band, *SEFI* Socioeconomic Factor Index, *Amb. phys* Ambulatory family physician, *Rx* prescription, *LOS* Length of stay

*All models control for maternal age; models 2 and 3 additionally control for regional of residence (refer to Additional file 5 for coefficients). Model 1: Miscarriage as a function of year, maternal age, and clinical covariates; Model 2: Miscarriage as a function of year, maternal age, and clinical+social covariates; Model 3: Miscarriage as a function of year, maternal age, and clinical+social+healthcare use covariates. All models use the binomial distribution and an identity link to obtain RDs

^a^Since 1984; ^b^In the 3 years before event; ^c^In the year before event; ^d^In the 2 years before event; ^e^In the 5 years before event; ^f^Reference age = 29; ^g^Reference region: Central; ^h^At time of event; ^i^For at least one month in the year before event; ^j^In $1000 increments; ^k^Over a 1-year period starting 2 years before the event date, ^l^In $100 increments

Model 2 includes clinical and social factors, including geographic region. Conditional on clinical risk factors, women living in more disadvantaged neighborhoods have elevated rates of miscarriage (RD = 0.01, 95% CI 0.004, 0.01), a 4 percentage point difference between the most and least disadvantaged neighborhoods. Women receiving income assistance have lower rates (RD = -0.02, 95% CI -0.03, − 0.02). While there is some variation across regions in rates of miscarriage, regions with higher rates do not share obvious sociodemographic characteristics.

Model 3 includes clinical, social, and health care use factors (Table 2). Conditional on clinical and social risk factors, women who are high/very high morbidity users of health care services are 12 percentage points (95% CI 0.09, 0.15), or two times, more likely to experience a miscarriage relative to women with low morbidity. This contrast is roughly equivalent to comparing pregnancy outcomes among women with asthma (RUB = 2) to women with two or more major morbidities (RUB = 4/5). Women who are healthy and moderate morbidity users of health care services are 3 (95% CI 0.02, 0.03) and 4 (95% CI 0.03, 0.04) percentage points more likely, respectively, to experience a miscarriage than women with low morbidity. Women with higher costs for hospital care in the year before the event are more likely to experience a miscarriage, but the magnitude is relatively small (RD = 0.004 per $1000, 95% CI 0.003, 0.006). Controlling for health care use factors does not change the associations between miscarriage and social risk factors, suggesting that the higher miscarriage rates in some geographic regions are not due to differential patterns of health care use. Controlling for health care use and social risk factors also does little to change the associations with clinical risk factors.

## Discussion

This analysis illustrates several important differences in the profiles of women who experience a first miscarriage and women who experience a live birth. Differences in clinical factors were particularly striking and robust to model specification, which suggests that the relationship between clinical predictors and miscarriage is not importantly confounded by social and health care use factors. The most important risk factor in the fully-adjusted model was morbidity (being in the high/very high category based on health care use in the previous two years): these women were twice as likely to experience a miscarriage compared to women with low morbidity. Conditional on clinical risk factors, social factors had moderate associations with miscarriage: women living in disadvantaged neighborhoods had higher rates of miscarriage while women on income assistance had lower rates. The latter finding is surprising since lower socioeconomic status is associated with risk factors for miscarriage. However, it is possible that women of higher socioeconomic status may be more likely to detect very early pregnancies, and therefore detect and seek care for early losses (for example, women in this group tend to be older and more likely to use fertility medications/treatment in order to conceive), which is important given our exposure definition. It is therefore possible that our sample underrepresents women with low income who experienced an early loss; as such, the reported association between income assistance and miscarriage should be interpreted with caution.

We estimate that 1 in 9 pregnant women in Manitoba experience and seek medical care for a miscarriage. Over the 2003–2014 period, we estimated an average annual rate of miscarriage of 11.3%. This estimate is similar to some [7], but not all [6, 21], studies using health care use data, and it is lower than estimates from preconception cohorts (25–31%) [28] since we identified events among pregnant women (as identified in administrative data) who seek medical care. These population-level rates should be reasonably accurate estimates of the number of women who seek medical care for a loss and they can be useful for health care providers and health care system planners.

Our exposure identification strategy is similar to approaches used in other studies that use diagnostic codes from health care use data. However, we improve on previous studies by adding physician billing and ED data to the typically used hospitalization data [6, 21, 26, 29]. This is important since miscarriages are increasingly treated in outpatient settings and many of the miscarriages we identified were not captured in the hospitalization data. We also improved the accuracy of our exposure identification strategy by using tariff codes in the physician billing data, which describe services provided, (Additional file 1), in conjunction with ICD codes.

The findings regarding the association between miscarriage and mental disorders add to a growing literature emphasizing an important connection between mental and physical health [30, 31], including research documenting the link between risk of suicidal behavior and physical disorders [32]. While one recent paper showed suicide attempts as an outcome of pregnancy loss [33], our findings suggest that a previous suicide attempt may be also associated with future miscarriages. This link may be due in part to the considerable morbidity and adversity associated with suicide attempts, including high rates of emergency department visits, violence, criminal victimization, and polypharmacy [34].

While administrative health data provide a comprehensive view of the population’s use of publicly-funded health care services, using it to estimate population rates of miscarriage may leave several gaps. Women who do not know they are pregnant, or who have no health care use with a pregnancy-related diagnosis, are not counted in our denominator, and women who do not seek any medical care for the miscarriage are not captured in our analysis. Little information exists on the probability of care-seeking after a miscarriage, but one study found that only 1–2% of Finnish women who had a miscarriage sought no medical care [35]. Women who only use services not covered by the public insurer (e.g., telephone consultations, psychological counseling by a non-physician provider) or not billed on a fee-for-service basis (e.g., consultations with nurses) will also not be captured. However, while we may not capture all services a woman who experiences a loss uses, our approach will capture any woman who uses at least one publicly insured service (and the vast majority of the Manitoba population is covered by the public insurer). The degree of undercounting may have lessened over our study period, as advances in early pregnancy detection have likely increased the share of miscarriages captured in administrative health data.

While we contribute to the existing evidence base by focusing on factors associated with first losses instead of recurrent losses, our findings may not be generalizable to the entire population given our exclusion of women with a previous therapeutic abortion (a substantial portion of the population; refer to Additional file 1 for additional details) and new Manitoba residents/immigrants who would not have been registered with Manitoba Health in the two years preceding the event. We also do not have information on behavioral risk factors such as smoking and alcohol consumption during pregnancy in our administrative databases for pregnancies that did not result in a live birth. While we include other factors that have not been previously examined in our analysis, it is possible that our estimated associations would change if we were able to control for the full set of established risk factors.

## Conclusion

Our findings suggest that past health care use and social factors may contribute important knowledge about women who experience a miscarriage, above and beyond the known clinical predictors. While attention to modifiable risk factors may help prevent some miscarriages, the current context of advanced maternal age, increased prevalence of comorbidities, and increased use of IVF suggests these events are likely to remain common. Awareness of, and attention to, factors associated with seeking medical care for a miscarriage by health care providers and administrators may help improve the patient-centeredness of the care these patients receive. A better understanding of which women are more likely to experience a loss may also contribute to better planning of health care system resources. Further research is needed to better understand the association of miscarriage with use of infertility drugs, a past suicide attempt, a diagnosis of endometriosis, and high morbidity.

## Supplementary Information

**Additional file 1.** Supplementary data and sample details (text description).

**Additional file 2.** Identified losses, by data source (table).

**Additional file 3.** Sample size as a function of exclusion criteria (table).

**Additional file 4.** Variable definitions (table).

**Additional file 5 **Multivariable analysis of clinical, social and health care use factors among women who experience their first miscarriage compared to women with a live birth: *all coefficients* (table).

**Additional file 6.** Global tests of significance (table).

## Data Availability

The datasets generated and analysed in the current study are not publicly available but may be requested from the Population Research Data Repository at the Manitoba Centre for Health Policy, University of Manitoba.

## Abbreviations

FP: Family physician
ED: Emergency department
RUB: Resource utilization band
RD: Risk difference
CI: Confidence interval

## Footnotes

1 For the purposes of this analysis, we define this as the first loss detectable in administrative data (these losses are therefore both detected and treated). Our goal here is to differentiate between first and recurrent losses. We discuss the nuances and limitations of this exposure definition later in the text.
