# Supplementary material for: Prevalence and clinical, social, and health care predictors of miscarriage
Source: BMC Pregnancy Childbirth. 2021 Mar 5;21:185. doi: 10.1186/s12884-021-03682-z (PMC7936485; doi:10.1186/s12884-021-03682-z)
Supplement: Supplementary file 1 — Additional file 1. Supplementary data and sample details (text description). [file 12884_2021_3682_MOESM1_ESM.docx]

**Additional file 1**

**Supplementary data and sample details**

Database details

International Classification of Disease (ICD) diagnostic codes are included in the physician visit (version 9) and hospitalization (version 10) records, as are tariff (procedure) codes that reflect the billable acts by physicians. Emergency department records (available only from 2009 forward, and only for the city of Winnipeg) include chief complaint codes and tariff codes. Filled prescriptions include the drug identification number (DIN).

Variable definitions

Ectopic pregnancy: at least one hospitalization or physician visit with a diagnosis of ectopic pregnancy ICD-9-CM 633 (including 633.1-2, 633.8-9), and ICD-10-CA O00 (including O00.0-2, O00.8, O00.9).

Miscarriage: at least one hospitalization or physician visit with a diagnosis of non-induced abortion ICD-9 631, 632, 634, 637; ICD-10 O02, O03, O06; or at least one ED visit with a chief complaint of “pregnancy issues” (either < or >20 weeks) and no delivery (live or stillborn) in the following 40 weeks. ED visits within +/-90 days of an ectopic pregnancy diagnosis were not included as miscarriages.

Due to the vague labels of some of the ICD-9 codes in the physician claims data (e.g., 637: unspecified abortion) and uncertainty about how they may be used by providers in this sensitive context, we combined the ICD-9 codes with tariff codes in the physician billing data to identify therapeutic vs. spontaneous abortions. 85% of “Legal abortions” (ICD-9 635) had an associated tariff code including the descriptor “therapeutic” (e.g., 34860 pregnancy and maternity, abortion (under 20 wks), therapeutic, by dilatation and curettage). “Spontaneous abortions” (ICD-9 634) either had no associated tariff code (59%) or had an associated tariff code with no mention of “therapeutic” (37%, e.g., 34855 pregnancy and maternity, abortion (under 20 wks), including dilatation and curettage). We therefore considered as therapeutic abortions claims with ICD-9 codes 631, 632, 634, 636, or 637 and one of 3 tariff codes indicating therapeutic abortion or therapeutic dilation and extraction (34860, 34861 or 34862). Claims with these ICD-9 codes and without one of these tariff codes were classified as miscarriages. Miscarriages within +/-60 days of a therapeutic abortion were excluded.

Deliveries: hospitalization with a diagnosis of a delivery (live and stillborn, singleton and multiple (ICD-9 V270-279, ICD-10 Z370-379)

Singleton live birth: hospitalization with a diagnosis of a singleton live birth (ICD-9 V270, ICD-10 Z370)

Stillbirth: one or more hospitalization with a diagnosis of stillbirth (ICD-9-CM V27.1, V27.3, V27.4, V27.6, V27.7; ICD-10-CA Z37.1, Z37.3, Z37.4, Z37.6, Z37.7)

Neonatal death: within 28 days of a live singleton birth

Infant death: within 365 days of a live singleton birth

Cohort definition

When comparing women who experience their first loss to those who have a live birth, we want to maximize the probability that women in neither group had experienced a previous loss. We therefore excluded from both groups women who: 1) were not continuously insured by Manitoba Health for at least two years prior to the loss or birth, 2) ever had a therapeutic abortion (as observable in our data from 1984-2014), and 3) had a previous loss (1984-2002) before the index loss or birth. Restrictions 2 and 3 are substantively important and move us away from examining the full population of women who experience a pregnancy. However they are important because therapeutic abortion may also be considered a type of loss^60^. After these exclusions, we therefore distinguish as clearly as possible women who experience their first ever loss (exposed) with women who have a live birth and no previous losses (unexposed).

Exposure identification by data source

We identified 25,714 miscarriages in the physician billing data, 13,680 in the hospitalization data, and 4,180 in the ED data. Since many losses appear in multiple data sources, this translates to 28,882 unique miscarriages over 10 years. 43.1% of the miscarriages identified in the physician billing data had a primary diagnosis of “Spontaneous abortion”, 31.2% of “Unspecified abortion” and 24.0% of “Missed abortion”. 58.3% of miscarriages identified in the hospitalization data had a primary diagnosis of “Spontaneous abortion” and 37.1% of “Other abnormal products of conception”. 98.2% of the miscarriages identified in the ED data had a chief complaint code of “Pregnancy Issues < 20 weeks”.

The chief complaint codes in the ED data are more vague than the ICD codes. To assess the validity of miscarriages identified in the ED data, we investigated what share of miscarriages identified in the ED were also observed in the inpatient and outpatient data. 45.8% of miscarriages identified in the ED data were not observed in the other two data sources (Table 1A). 47.8% of miscarriages identified in physician billing records and 8.4% of miscarriages identified in hospitalization records are only observed in those respective data sources. Among all identified miscarriages, 46.8% were observed in at least 2 data sources (Table A1). For ectopic pregnancies, 34.4% are observed in both the outpatient and inpatient data, while 62.2% are observed only in the physician claims data.
