# Supplementary material for: Prevalence and clinical, social, and health care predictors of miscarriage
Source: BMC Pregnancy Childbirth. 2021 Mar 5;21:185. doi: 10.1186/s12884-021-03682-z (PMC7936485; doi:10.1186/s12884-021-03682-z)
Supplement: Supplementary file 2 — Additional file 2. Identified losses, by data source (table). [file 12884_2021_3682_MOESM2_ESM.docx]

**Additional file 2: Identified losses, by data source**

| **Data source** | **All identified miscarriages (n=28882)** | | **All identified ectopic (n=6410)** | | **ED-identified miscarriages (n=4180)** | |
| --- | --- | --- | --- | --- | --- | --- |
|  | **n** | **%** | **n** | **%** | **n** | **%** |
| MD claims only | 12304 | 42.6 | 3989 | 62.2 | - | - |
| Hospital claims only | 1148 | 4.0 | 216 | 3.4 | - | - |
| MD+hospital claims | 11250 | 39.0 | 2205 | 34.4 | - | - |
| ED claims only | 1916 | 6.6 | - | - | 1916 | 45.8 |
| MD+ED claims | 982 | 3.4 | - | - | 982 | 23.5 |
| Hospital+ED claims | 104 | 0.4 | - | - | 104 | 2.5 |
| Reported in all 3 sources | 1178 | 4.1 | - | - | 1178 | 28.2 |
|  |  |  |  |  |  |  |
| MD: medical; ED: emergency department | | | | | | |
