# Supplementary material for: Prevalence and clinical, social, and health care predictors of miscarriage
Source: BMC Pregnancy Childbirth. 2021 Mar 5;21:185. doi: 10.1186/s12884-021-03682-z (PMC7936485; doi:10.1186/s12884-021-03682-z)
Supplement: Supplementary file 3 — Additional file 3. Sample size as a function of exclusion criteria (table). [file 12884_2021_3682_MOESM3_ESM.docx]

**Additional file 3 – Sample size as a function of exclusion criteria**

|  | | | |  |
| --- | --- | --- | --- | --- |
|  |  | **Total** | **Loss cohort** | **Live birth cohort** |
| Initial sample | | 168,639 | 27,572 | 141,067 |
| Exclusion criteria: | |  |  |  |
|  | Previous documented loss (n, %)^a^ | 31,508 (18.6%) | 900 (3.3%) | 30,608 (21.7%) |
|  | Previous therapeutic abortion (n, %) | 42,496 (25.2%) | 8,965 (32.5%) | 33,531 (23.8%) |
|  | Exclude subsequent loss^b^ | 2,783 (1.7%) | 2,783 (10.1%) | - |
|  | Not in the 2-year wash-in (n, %)^c^ | 10,074 (6.0%) | 1,893 (6.9%) | 8,181 (5.8%) |
| Final sample | | 81,778 | 13,031 | 68,747 |
| % reflects percent of column total ^a^Includes documented miscarriages, ectopic pregnancies, infant deaths, and stillbirths prior to the index birth/loss (1984-2002). Stillbirths are defined as one or more hospitalization with a diagnosis of stillbirth (ICD-9-CM V27.1, V27.3, V27.4, V27.6, V27.7; ICD-10-CA Z37.1, Z37.3, Z37.4, Z37.6, Z37.7). Neonatal death is within 28 days, and infant death within 365 days, of a live singleton birth; ^b^Losses following the index event; ^c^Women not registered with Manitoba Health for two years prior to the loss/birth | | | | |
