# Supplementary material for: Prevalence and clinical, social, and health care predictors of miscarriage
Source: BMC Pregnancy Childbirth. 2021 Mar 5;21:185. doi: 10.1186/s12884-021-03682-z (PMC7936485; doi:10.1186/s12884-021-03682-z)
Supplement: Supplementary file 4 — Additional file 4. Variable definitions (table). [file 12884_2021_3682_MOESM4_ESM.docx]

**Additional file 4 – Variable definitions**

|  | **Variable** | **Description** | **Timeframe^a^** | **Levels** |
| --- | --- | --- | --- | --- |
| **Clinical factors** | | | | |
|  | Parity | Nulliparous, primiparous, or multiparous based on total number of live or stillborn births | 1984-2014 | 0, 1, 2 |
|  | Previous c-section | Incident c-section or diagnosis indicating a previous c-section for any live or stillborn deliveries | 1984-2014 | 0 (no), 1(yes) |
|  | Hypertension | 1+ MD visit, 1+ hospitalization, or 2+ relevant prescriptions (excluding pregnancy-induced hypertension) | Past year | 0 (no), 1(yes) |
|  | Diabetes | 2+ MD visits, 1+ hospitalization, or 1+ relevant prescriptions (excluding gestational diabetes) | Past 3 years | 0 (no), 1(yes) |
|  | Infertility drugs | 1+ prescription for ovulation induction or controlled ovarian hyperstimulation | Past 2 years | 0 (no), 1(yes) |
|  | Endometriosis | 2+ MD visits or 1+ hospitalization | Past 3 years | 0 (no), 1(yes) |
|  | Substance abuse | 1+ MD visit or 1+ hospitalization | Past 2 years | 0 (no), 1(yes) |
|  | Suicide attempt | 1+ hospitalization for suicide or self-inflicted injury | Past 5 years | 0 (no), 1(yes) |
|  | Mood or anxiety disorders | *Includes any of the following:* | Past 2 years | 0 (no), 1(yes) |
|  |  | - One or more hospitalizations with a diagnosis of depressive disorder, affective psychoses, neurotic depression or adjustment reaction, anxiety disorders, anxiety states, phobic disorders, or obsessive-compulsive disorders | | |
|  |  | - One or more MD visits with a diagnosis of depressive disorder, affective psychoses, or adjustment reaction | | |
|  |  | - One or more MD visits with a diagnosis of anxiety disorders AND 1+ prescriptions for an antidepressant or mood stabilizer | | |
|  |  | - 3+ MD visits with a diagnosis of anxiety disorders | | |
| **Social factors** | | | | |
|  | Socio-Economic Factor Index (SEFI) | Reflects average household income, percent of single parent households, the unemployment rate, and the high school education rate in the mother's Census Dissemination Area (negative values are favorable) | At time of event | Continuous, centered on 0 |
|  | Region | 9 rural geographic areas, 12 urban Community Areas inside Winnipeg^b^ | At time of event | Categorical |
|  | Income assistance | Receipt of IA for at least one month | Past year | 0 (no), 1 (yes) |
| **Health care use factors^c^** | | | | |
|  | Inpatient costs | Hospitalization costs, in 2010 dollars | Past year | Continuous |
|  | Inpatient LOS | Length of hospitalization, in days | Past year | Integer |
|  | Ambulatory care costs | Ambulatory care costs, in 2010 dollars | Past year | Continuous |
|  | Ambulatory care visits | Number of visits to all ambulatory care providers | Past year | Integer |
|  | Psychotropic medications: incidence | Any new psychotropic medications prescribed in year before event | Over a 1-year period in past 2 years | 0 (no), 1(yes) |
|  | Psychotropic medications: count | Number of psychotropic medications prescribed in year before event | Over a 1-year period in past 2 years | Integer |
|  | Psychotropic medications: costs | Total cost of psychotropic medications in year before event | Over a 1-year period in past 2 years | Continuous |
|  | Resource utilization band (RUB) | Resource utilization band, regrouped slightly for the purposes of this analysis: non-users, healthy users, low morbidity, moderate morbidity, high/very high morbidity | Past year | 0, 1, 2, 3, 4 |
| ^a^Time with respect to event (e.g. "Past 2 years" reflects the two years leading up to the birth or loss); ^b^Custom cut of regional data. Citation: Statistics Canada. 2011. Profile of the population, Custom Geography Profile. National Household Survey of Canada (database). Community Data Program (distributor). Date released Wednesday, September 11, 2013. Communitydata.ca (accessed April 13, 2016); ^c^All measures exclude pregnancy-related care, including prenatal care and delivery. | | | | |
|  |  |  |  |  |
