# Supplementary material for: Prevalence and clinical, social, and health care predictors of miscarriage
Source: BMC Pregnancy Childbirth. 2021 Mar 5;21:185. doi: 10.1186/s12884-021-03682-z (PMC7936485; doi:10.1186/s12884-021-03682-z)
Supplement: Supplementary file 5 — Additional file 5 Multivariable analysis of clinical, social and health care use factors among women who experience their first miscarriage compared to women with a live birth: all coefficients (table). [file 12884_2021_3682_MOESM5_ESM.docx]

| **Additional file 5 - Multivariable analysis of clinical, social and health care use factors among women who experience their first EPM compared to women with a live birth: *all coefficients*** | | | | | | | |
| --- | --- | --- | --- | --- | --- | --- | --- |
|  |  |  |  |  |  |  |  |
| **Variable** | | **Model 1: RD (95%CI) *n*=81,778** |  | **Model 2: RD (95%CI) *n*=81,664** |  | **Model 3: RD (95%CI) *n*=81,664** |  |
| Year of event | | 0.001 (-0.000, 0.002) |  | 0.001 (0.000, 0.002) |  | 0.001 (0.0001, 0.002) |  |
| **Clinical** | |  |  |  |  |  |  |
| Parity^a^ | | | | | | | |
|  | Primiparous vs. nulliparous | -0.04 (-0.04, -0.03) |  | -0.03 (-0.04, -0.03) |  | -0.03 (-0.04, -0.02) |  |
|  | Multiparous vs. nulliparous | -0.03 (-0.04, -0.03) |  | -0.03 (-0.04, -0.03) |  | -0.03 (-0.04, -0.02) |  |
| Previous c-section^a^ | | 0.01 (0.004, 0.02) |  | 0.01 (0.004, 0.02) |  | 0.01 (0.0002, 0.02) |  |
| Diabetes^b^ | | 0.01 (-0.0001, 0.03) |  | 0.01 (-0.005, 0.02) |  | -0.003 (-0.02, 0.01) |  |
| Endometriosis^b^ | | 0.05 (0.02, 0.09) |  | 0.05 (0.02, 0.09) |  | 0.04 (0.01 0.08) |  |
| Hypertension^c^ | | -0.01 (-0.02, 0.01) |  | -0.01 (-0.02, 0.005) |  | -0.02 (-0.04, -0.01) |  |
| Infertility drug use^d^ | | 0.05 (0.04, 0.07) |  | 0.06 (0.04, 0.08) |  | 0.05 (0.03, 0.07) |  |
| Mood or anxiety disorders^d^ | | 0.05 (0.04, 0.05) |  | 0.05 (0.04, 0.06) |  | 0.03 (0.02, 0.04) |  |
| Substance abuse^d^ | | 0.01 (-0.01, 0.03) |  | 0.01 (-0.01, 0.02) |  | -0.01 (-0.03, 0.01) |  |
| Suicide attempt^e^ | | 0.06 (0.02, 0.09) |  | 0.05 (0.01, 0.08) |  | 0.04 (0.001, 0.07) |  |
| Maternal age at event^f^ | | | | | | | |
|  | 11-14 | 0.14 (0.07, 0.21) |  | 0.14 (0.07, 0.21) |  | 0.14 (0.08, 0.21) |  |
|  | 15 | 0.06 (0.02, 0.10) |  | 0.06 (0.02, 0.10) |  | 0.06 (0.02, 0.10) |  |
|  | 16 | 0.03 (0.01, 0.06) |  | 0.03 (0.00, 0.06) |  | 0.03 (0.001, 0.06) |  |
|  | 17 | 0.02 (0.00, 0.04) |  | 0.02 (-0.01, 0.04) |  | 0.02 (-0.01, 0.04) |  |
|  | 18 | 0.04 (0.02, 0.06) |  | 0.03 (0.01, 0.05) |  | 0.03 (0.01, 0.05) |  |
|  | 19 | 0.01 (0.00, 0.03) |  | 0.01 (0.00, 0.03) |  | 0.01 (-0.01, 0.03) |  |
|  | 20 | 0.01 (-0.01, 0.03) |  | 0.01 (-0.01, 0.02) |  | 0.01 (-0.01, 0.02) |  |
|  | 21 | 0.00 (-0.02, 0.01) |  | -0.003 (-0.02, 0.01) |  | -0.002 (-0.02, 0.01) |  |
|  | 22 | 0.01 (-0.01, 0.02) |  | 0.008 (-0.01, 0.02) |  | 0.01 (-0.01, 0.02) |  |
|  | 23 | 0.00 (-0.02, 0.01) |  | 0.00 (-0.02, 0.01) |  | -0.0001 (-0.02, 0.02) |  |
|  | 24 | 0.00 (-0.01, 0.02) |  | 0.004 (-0.01, 0.02) |  | 0.003 (-0.01, 0.02) |  |
|  | 25 | 0.00 (-0.01, 0.02) |  | 0.004 (-0.01, 0.02) |  | 0.003 (-0.01, 0.02) |  |
|  | 26 | 0.01 (0.00, 0.02) |  | 0.011 (0.00, 0.02) |  | 0.01 (-0.005, 0.02) |  |
|  | 27 | 0.00 (-0.02, 0.01) |  | -0.003 (-0.02, 0.01) |  | -0.003 (-0.02, 0.01) |  |
|  | 28 | -0.01 (-0.02, 0.01) |  | -0.005 (-0.02, 0.01) |  | -0.005 (-0.02, 0.009) |  |
|  | 30 | -0.01 (-0.02, 0.00) |  | -0.01 (-0.02, 0.00) |  | -0.008 (-0.02, 0.01) |  |
|  | 31 | 0.00 (-0.02, 0.01) |  | -0.002 (-0.02, 0.01) |  | -0.001 (-0.02, 0.01) |  |
|  | 32 | 0.00 (-0.01, 0.02) |  | 0.003 (-0.01, 0.02) |  | 0.004 (-0.01, 0.02) |  |
|  | 33 | 0.00 (-0.02, 0.01) |  | 0.00 (-0.01, 0.01) |  | -0.001 (-0.02, 0.01) |  |
|  | 34 | 0.02 (0.01, 0.04) |  | 0.02 (0.01, 0.04) |  | 0.02 (0.004, 0.04) |  |
|  |  |  |  |  |  |  |  |
|  |  |  |  |  |  |  |  |
| **Variable** | | **Model 1: RD (95%CI) *n*=81,778** |  | **Model 2: RD (95%CI) *n*=81,664** |  | **Model 3: RD (95%CI) *n*=81,664** |  |
| Maternal age at event^f^ | | | | | | | |
|  | 35 | 0.04 (0.02, 0.05) |  | 0.04 (0.02, 0.05) |  | 0.04 (0.02, 0.05) |  |
|  | 36 | 0.04 (0.02, 0.06) |  | 0.04 (0.02, 0.06) |  | 0.04 (0.02, 0.06) |  |
|  | 37 | 0.05 (0.03, 0.07) |  | 0.05 (0.03, 0.07) |  | 0.05 (0.03, 0.07) |  |
|  | 38 | 0.06 (0.03, 0.08) |  | 0.06 (0.03, 0.08) |  | 0.05 (0.03, 0.08) |  |
|  | 39 | 0.10 (0.07, 0.13) |  | 0.10 (0.07, 0.13) |  | 0.09 (0.06, 0.12) |  |
|  | 40 | 0.12 (0.09, 0.15) |  | 0.12 (0.09, 0.15) |  | 0.11 (0.08, 0.15) |  |
|  | 41 | 0.18 (0.14, 0.23) |  | 0.18 (0.14, 0.23) |  | 0.17 (0.13, 0.22) |  |
|  | 42 | 0.20 (0.14, 0.25) |  | 0.20 (0.14, 0.25) |  | 0.19 (0.14, 0.25) |  |
|  | 43 | 0.28 (0.21, 0.35) |  | 0.28 (0.21, 0.35) |  | 0.27 (0.20, 0.33) |  |
|  | 44 | 0.37 (0.28, 0.46) |  | 0.37 (0.27, 0.46) |  | 0.35 (0.25, 0.44) |  |
|  | 45 | 0.37 (0.25, 0.48) |  | 0.37 (0.25, 0.49) |  | 0.36 (0.24, 0.47) |  |
|  | 46+ | 0.57 (0.49, 0.66) |  | 0.58 (0.49, 0.66) |  | 0.55 (0.46, 0.63) |  |
| **Social** | |  |  |  |  |  |  |
| Mother's SEFI^g^ | |  |  | 0.01 (0.002, 0.006) |  | 0.01 (0.007, 0.013) |  |
| Income assistance^h^ | |  |  | -0.02 (-0.03, -0.01) |  | -0.03 (-0.04, -0.02) |  |
| Mother's region: rural^i^ | | | | | | | |
|  | North Eastman |  |  | 0.01 (-0.01, 0.02) |  | 0.01 (-0.01, 0.02) |  |
|  | South Eastman |  |  | 0.00 (-0.01, 0.01) |  | 0.001 (-0.01, 0.01) |  |
|  | Interlake |  |  | 0.00 (-0.01, 0.01) |  | 0.001 (-0.01, 0.01) |  |
|  | Nor-Man |  |  | 0.01 (-0.01, 0.03) |  | 0.01 (-0.002, 0.03) |  |
|  | Parkland |  |  | 0.01 (0.00, 0.03) |  | 0.003 (-0.01, 0.02) |  |
|  | Burntwood/Churchill |  |  | 0.02 (0.00, 0.03) |  | 0.02 (0.01, 0.04) |  |
|  | Brandon |  |  | 0.03 (0.01, 0.04) |  | 0.02 (0.003, 0.03) |  |
|  | Assiniboine |  |  | 0.01 (0.00, 0.02) |  | 0.01 (-0.01, 0.02) |  |
| Mother's region: urban/Winnipeg^h^ | | | | | | | |
|  | St. James-Assiniboia |  |  | 0.02 (0.01, 0.04) |  | 0.02 (0.003, 0.04) |  |
|  | Assiniboine South |  |  | 0.02 (0.00, 0.03) |  | 0.01 (-0.01, 0.03) |  |
|  | Fort Garry |  |  | 0.03 (0.01, 0.04) |  | 0.02 (0.01, 0.04) |  |
|  | St. Vital |  |  | 0.04 (0.02, 0.05) |  | 0.03 (0.02, 0.05) |  |
|  | St. Boniface |  |  | 0.01 (0.00, 0.02) |  | 0.01 (-0.01, 0.02) |  |
|  | Transcona |  |  | 0.01 (-0.01, 0.03) |  | 0.01 (-0.01, 0.03) |  |
|  | River East |  |  | 0.01 (0.00, 0.02) |  | 0.01 (-0.002, 0.02) |  |
|  | Seven Oaks |  |  | 0.00 (-0.01, 0.01) |  | -0.001 (-0.02, 0.01) |  |
|  | Inkster |  |  | 0.00 (-0.02, 0.01) |  | -0.004 (-0.02, 0.01) |  |
|  | Point Douglas |  |  | 0.00 (-0.01, 0.02) |  | 0.002 (-0.01, 0.02) |  |
|  | Downtown |  |  | 0.03 (0.02, 0.04) |  | 0.02 (0.01, 0.04) |  |
|  | River Heights |  |  | 0.03 (0.01, 0.04) | <0.01 | 0.03 (0.01, 0.04) | <0.01 |
|  |  |  |  |  |  |  |  |
| **Variable** | | **Model 1: RD (95%CI) *n*=81,778** |  | **Model 2: RD (95%CI) *n*=81,664** |  | **Model 3: RD (95%CI) *n*=81,664** |  |
| **Health care use** | |  |  |  |  |  |  |
| RUB^c^ | | | | | | | |
|  | 0 vs. 2 |  |  |  |  | -0.03 (-0.04, -0.03) |  |
|  | 1 vs. 2 |  |  |  |  | 0.03 (0.02, 0.04) |  |
|  | 3 vs. 2 |  |  |  |  | 0.04 (0.03, 0.05) |  |
|  | 4+ vs. 2 |  |  |  |  | 0.14 (0.11, 0.17) |  |
| Hospitalization costs (2010$)^c, k^ | |  |  |  |  | 0.005 (0.003, 0.006) |  |
| Amb. phys. costs (2010$)^c, k^ | |  |  |  |  | 0.01 (-0.01, 0.03) |  |
| Psychotropic Rx costs (2010$)^j, l^ | |  |  |  |  | -0.002 (-0.004, 0.0001) |  |
|  |  |  |  |  |  |  |  |
| EPM: Miscarriage and ectopic pregnancy; RD: risk difference; CI: confidence interval; RUB: Resource Utilization Band; SEFI: Socioeconomic Factor Index; Amb. phys: Ambulatory physician; Rx: prescription; LOS: Length of stay | | | | | | | |
| Model 1: EPM as a function of year, maternal age, and clinical covariates; Model 2: EPM as a function of year, maternal age, and clinical+social covariates; Model 3: EPM as a function of year, maternal age, and clinical+social+healthcare use covariates. All models use the binomial distribution and an identity link to obtain RDs. | | | | | | | |
| ^a^Since 1984; ^b^In the 3 years before event; ^c^In the year before event; ^d^In the 2 years before event; ^e^In the 5 years before event; ^f^Reference age=29; ^g^At time of event; ^h^For at least one month in the year before event; ^i^Reference region: Central; ^j^Over a 1-year period starting 2 years before the event date, ^k^In $1000 increments; ^l^In $100 increments | | | | | | | |
