# Supplementary material for: Prevalence and clinical, social, and health care predictors of miscarriage
Source: BMC Pregnancy Childbirth. 2021 Mar 5;21:185. doi: 10.1186/s12884-021-03682-z (PMC7936485; doi:10.1186/s12884-021-03682-z)
Supplement: Supplementary file 6 — Additional file 6. Global tests of significance (table). [file 12884_2021_3682_MOESM6_ESM.docx]

| **Additional file 6: Global tests of significance** | | | | | | |
| --- | --- | --- | --- | --- | --- | --- |
|  | **Model 1: n=81,778** | | **Model 2: n=81,664** | | **Model 3: n=81,664** | |
| **Variable** | **X^2^** | **p-value** | **X^2^** | **p-value** | **X^2^** | **p-value** |
| Year of event | 3.77 | 0.05 | 4.01 | 0.04 | 4.53 | 0.03 |
| Maternal age at event | 804.76 | <0.01 | 783.35 | <0.01 | 579.75 | <0.01 |
| Parity | 162.39 | <0.01 | 137.42 | <0.01 | 92.03 | <0.01 |
| Previous c-section | 8.33 | <0.01 | 8.65 | <0.01 | 4.04 | 0.04 |
| Hypertension | 1.4 | 0.24 | 1.63 | 0.20 | 9.48 | <0.01 |
| Diabetes | 3.98 | 0.05 | 1.77 | 0.18 | 0.11 | 0.74 |
| Infertility drug use | 36.39 | <0.01 | 40.06 | <0.01 | 25.08 | <0.01 |
| Endometriosis | 9.38 | <0.01 | 10.29 | <0.01 | 5.00 | 0.03 |
| Substance abuse | 1.69 | 0.19 | 0.62 | 0.43 | 0.78 | 0.38 |
| Suicide attempt | 11.42 | <0.01 | 8.53 | <0.01 | 4.13 | 0.04 |
| Mood or anxiety disorders | 140.81 | <0.01 | 151.4 | <0.01 | 38.9 | <0.01 |
| SEFI |  |  | 34.66 | <0.01 | 36.76 | <0.01 |
| Mother's region |  |  | 72.02 | <0.01 | 58.08 | <0.01 |
| Income assistance |  |  | 23.72 | <0.01 | 39.51 | <0.01 |
| RUB |  |  |  |  | 391.12 | <0.01 |
| Amb. phys. costs (2010$) |  |  |  |  | 0.73 | 0.39 |
| Hospitalization costs (2010$) |  |  |  |  | 52.99 | <0.01 |
| Psychotropic Rx costs (2010$) |  |  |  |  | 3.48 | 0.06 |
